# 3D MRI Tract‐Specific Spinal Cord Lesion Pattern Improves Prediction of Distinct Neurological Recovery

**DOI:** 10.1002/acn3.70087

**Published:** 2025-06-27

**Authors:** Lynn Farner, Tim M. Emmenegger, Simon Schading‐Sassenhausen, Julia Berroth, Maryam Seif, Armin Curt, Patrick Freund, The Nogo Inhibition in Spinal Cord Injury Study Group

**Affiliations:** ^1^ Spinal Cord Injury Center, Balgrist University Hospital University of Zurich Zurich Switzerland; ^2^ Department of Biomedical Imaging and Image‐Guided Therapy, High Field MR Center Medical University of Vienna Vienna Austria; ^3^ Department of Neurophysics Max Planck Institute for Human Cognitive and Brain Sciences Leipzig Germany; ^4^ Department of Imaging Neuroscience, UCL Queen Square Institute of Neurology University College London London UK

**Keywords:** lesion segmentation, MRI, recovery, spinal cord injury

## Abstract

**Objective:**

To distinguish lateralized motor‐ and sensory‐tract damage after acute spinal cord injury (SCI) and explore its predictive power for motor and sensory recovery.

**Methods:**

Thirty‐five SCI patients (two female) from a multi‐center data set (placebo‐arm of the Nogo‐A‐Inhibition in SCI trial) underwent routine T2‐weighted sagittal MRI scans at the lesion site at baseline (19.9 days, 95% confidence interval [CI]: 17.9–21.8), 1‐month (54.2 days, 95% CI: 52.1–56.2), and 6‐month (192.4 days, 95% CI: 181.3–203.6) post‐injury. Concurrently with the MRI scans, clinical examinations were performed. Lesions were manually segmented across all slices, and 3D‐tract damage was assessed by determining the overlap between segmented lesions and identified motor and sensory tracts in the axial plane. The relationship between lesion assessments and baseline‐adjusted clinical outcomes at 6 months was explored.

**Results:**

Over the 6‐month, patients recovered by 4.95 motor points/month (95% CI: 3.89–5.89, *p* < 0.001) on the International Standards for the Neurological Classification of SCI scale, 2.28 light‐touch points/month (95% CI: 1.43–3.12, *p* < 0.001), and 2.06 pinprick points/month (95% CI: 1.21–2.91, *p* < 0.001). Lesion volume decreased from 381.82mm ^3^ (95% CI: 295.78–467.87) by −14.04 mm^3^/month (95% CI: −25.39 to −1.56, *p* = 0.023). MRI visible changes in motor tract damage over the 6‐month were marginal (0.02%/month, 95% CI: −0.81 to −1.02, *p* = 0.971). Changes in the sensory tracts were more pronounced, decreasing by −0.69%/month (95% CI: −1.29 to −0.09, *p* = 0.05). Left‐and‐right motor‐tract damage at baseline significantly predicted left‐and‐right motor score recovery (*R* ^2^ = 0.75, *p* = 0.015), while baseline left‐and‐right sensory‐tract damage significantly predicted improvements in left‐and‐right pin‐prick scores (*R* ^2^ = 0.79, *p* = 0.024).

**Interpretation:**

Revealing the extent of damage to spinal motor‐and sensory‐pathways early after SCI is a valuable predictor of related neurological recovery. Tracking 3D dynamics of major spinal pathways has the potential to enhance diagnostic accuracy and patient stratification for future clinical trials.

## 1 Introduction

Traumatic spinal cord injury (SCI) has devastating clinical consequences, as recovery in most individuals is limited [1, 2]. In approximately 70% of SCI patients, the lesion is anatomically incomplete, a concept previously explored using tissue bridges (TB) [3, 4]. TB are defined as spared tissue surrounding the post‐traumatic cyst, visible on the midsagittal slice of a T2‐weighted sagittal MRI. Ventral TB, located anterior to the cyst, are associated with motor function and predict motor recovery, while dorsal TB, located posterior to the cyst, are predictive of sensory recovery [5, 6, 7, 8, 9]. However, their utility is limited because they rely on two‐dimensional (2D) evaluations of the lesion, which do not capture the full extent of the lesion or its precise location in the spinal cord. This can significantly impact clinical recovery, especially in cases where the lesion is not exactly midsagittal or is not symmetrically along the midsagittal axis. Lesions can be classified based on their location within the spinal cord—central, ventral, dorsal, or complete—using the presence of TB. This classification provides important insights into recovery patterns based on the involvement of different tracts. However, this remains a 2D assessment. Developing clinically applicable 3D MRI biomarkers to quantify tract damage at the lesion site could overcome the spatial limitations of 2D lesion reconstruction. This multicenter, retrospective study aimed to enhance lesion assessment by analyzing 3D tract‐specific damage using clinically routine sagittal T2‐weighted MRI scans. An earlier study has shown that spinal tract damage assessed on axial MRI scans can predict clinical recovery [10]. Unlike most axial scans, which rely on gradient echo sequences, sagittal turbo spin‐echo T2‐weighted imaging is less susceptible to artifacts, ensuring more accurate lesion segmentations [11, 12]. The clinical validity and potential of tract damage as a predictive biomarker is then compared to the conventional lesion biomarkers assessed on sagittal MRI scans, such as TB and lesion volume [4, 5, 6, 7, 13, 14, 15].

## 2 Methods

### 2.1 Patients and Study Design

This study includes MRI data from 48 placebo‐treated patients enrolled in the phase 2b NISCI clinical trial conducted from May 2019 to July 2022 at multiple European centers [16]. The trial included adult patients (18–70 years) with acute cervical SCI (C1–C8), enrolled within 4–28 days post‐injury. Patients underwent surgery before trial inclusion, and each site followed its standard SCI rehabilitation protocol for up to 6 months after the injury. Participants were selected using the Unbiased Recursive Partitioning (URP) method, which excluded those predicted to reach maximal UEMS recovery to avoid a ceiling effect, and were randomized in a 2:1 ratio to receive either NG101 or placebo. This analysis includes only those who received intrathecal placebo (phosphate‐buffered saline) at the L3/L4 level.

Patients underwent MRI scans at three time points: baseline (19.9 days, 95% confidence interval [CI]: 17.9–21.8), 1‐month (54.2 days, 95% CI: 52.1–56.2), and 6‐month (192.4 days, 95% CI: 181.3–203.6) post‐injury (Table 1). Patient numbers vary across time points due to differences in clinical stability and availability. All available data was included at each time point to maximize statistical power in cross‐sectional analyses, and variable time point availability was accounted for in longitudinal linear mixed‐effects models. MRI parameters are reported in Table S1. Exclusion criteria included the presence of artifacts in the cervical region, missing data, and non‐definable lesions, resulting in the exclusion of 13 subjects in this study. The final cohort comprised 35 subjects, of whom 2 were female.

**TABLE 1:** Clinical and epidemiologic data for all study subjects for all three‐time points baseline, 1‐ and 6‐month time points.

Characteristics	Study population (*n* = 35)
Age at time of injury (years)	47.5 (95% CI: 41.2–53.9)
Sex, *n*, (%)	Female, 2, (5.7) Male, 33, (94.3)

Characteristics	Study population at baseline (*n* = 28)	Study population at 1‐month follow up (*n* = 30)	Study population at 6‐month follow up (*n* = 33)
AIS *n*, (%)			
A	5 (17.9)	2 (6.7)	2 (6.1)
B	6 (21.4)	6 (20.0)	6 (18.2)
C	10 (35.7)	8 (26.7)	4 (12.1)
D	7 (25.0)	14 (46.7)	20 (60.6)
E	0 (0)	0 (0)	1 (3.0)
NLI *n*, (%)			
C2	3 (10.7)	3 (10.0)	3 (9.1)
C3	7 (25.0)	2 (6.7)	3 (9.1)
C4	10 (35.7)	10 (33.3)	12 (36.4)
C5	7 (25.0)	12 (40.0)	5 (15.2)
C6	1 (3.6)	3 (10.0)	6 (18.2)
C7	0 (0)	0 (0)	2 (6.1)
C8	0 (0)	0 (0)	1 (3.0)
INT	0 (0)	0 (0)	1 (3.0)
Time interval between injury and MRI (days)	19.9 (95% CI: 17.9–21.8)	54.2 (95% CI: 52.1–56.2)	192.4 (95% CI: 181.3–203.6)

### 2.2 Standard Protocol Approvals, Registrations, and Patient Consents

The local ethics committees approved the study protocol (ClinicalTrials.gov, Identifier: NCT03935321), and all SCI patients gave informed, written consent before study enrollment.

### 2.3 Clinical Assessments

SCI patients underwent clinical evaluations following the International Standards for Neurological Classification of SCI (ISNCSCI) examination concurrently with the MRI scans [17]. These evaluations include motor scores, pinprick, and light touch, and the American Spinal Injury Association Impairment Scale (AIS) grade. Clinical scores were normalized by including only those measured below the neurological level of injury (NLI) and dividing them by the maximum possible score. This approach captures variability in recovery based on the NLI [9]. To evaluate recovery relative to initial impairment, clinical score changes were calculated as the difference between scores at 1‐ and 6‐month time points [6]. Since these are delta scores, normalization was not necessary, as the lesion level is inherently considered through the score changes over time.

### 2.4 Image Analysis

Sagittal turbo spin‐echo T2‐weighted scans were used to analyze the lesion, defined as the hyperintense intramedullary region, and manually segmented on all slices. A single rater (LF), blinded to patient identity and time point, performed the segmentation using Jim software (version 7.0, Xinapse Systems). Lesion segmentation was done across all slices where the lesion was present and obtained as a soft volume, representing the proportion of a voxel occupied by the segmented region of interest (ROI). ROI values ranged from 0 to 1, where 0 indicated no lesion presence and 1 indicated the voxel was fully occupied by the lesion.

The spinal cord was automatically segmented using the *seg_sc_lesion_t2w_sci* task of the *sct_deepseg* function in the Spinal Cord Toolbox (version 6.2) [15, 18]. All segmentations were reviewed and manually corrected when necessary. Both lesion and spinal cord segmentations were registered and warped into the PAM50 template space [19].

To analyze the impact of the lesion on the white matter tracts of the spinal cord, we used a custom MATLAB (version 2024a) script to map the segmented lesion volume onto axial, coronal, and sagittal planes (code available under https://github.com/NeuroimagingBalgrist).

**FIGURE 1:**
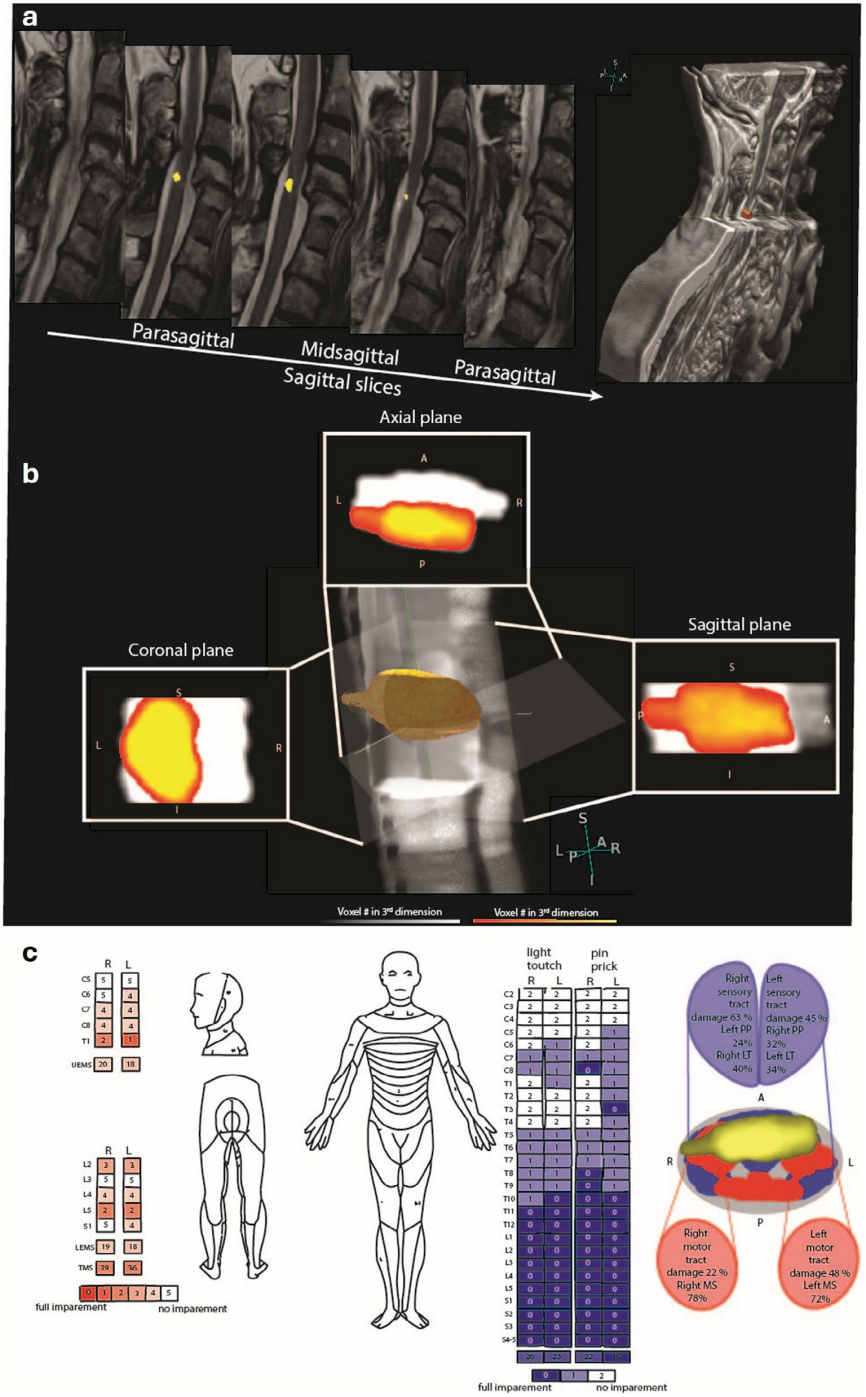
Illustration of a patient example of the 3D lesion segmentation and tract damage analysis. (a) T2‐weighted sagittal slices were observed where the hyperintensity of the lesion is segmented (yellow), providing the lesion volume. (b) A glass‐spine view projects the lesion into sagittal, axial, and coronal planes, creating a 3D representation. (c) This shows the ISNCSCI examination and lesion overlap comparison, revealing greater impairment in the left motor tracts, consistent with lower motor scores on the left side in the ISNCSCI assessment. In contrast, the sensory tracts show more damage on the right side, corresponding to lower pinprick scores on the left. An axial view illustrates the spinal cord motor white matter tracts (shown in red), sensory white matter tracts (in blue), alongside the lesion (in yellow), with the overlap between the tracts and the lesion quantified as a percentage of damage.

The same procedure was applied to the segmented spinal cord, resulting in a three‐dimensional (3D) representation. To retain 3D information in each of the maps, voxel values in each plane of the lesion map reflected the lesion's extent in the third, through‐plane dimension. This approach allows for a comprehensive spatial understanding of the lesion's influence on the spinal cord. The resulting 3D representation is defined by the lesion's superior–inferior boundaries and the spinal cord's anterior–posterior boundaries. A patient example of the 3D lesion segmentation can be seen in Figure 1.

### 2.5 White‐Matter Tract Atlas

To evaluate the specific white‐matter (WM) tract damage in the axial plane, masks of the relevant sensory (fasciculus gracilis, fasciculus cuneatus, ventral spinocerebellar tract, spinothalamic, spinoreticular, and spino‐olivary tract) and motor (lateral and ventral corticospinal tract, rubrospinal tract, lateral and ventral reticulospinal tract, lateral and ventral vestibulospinal tract, tectospinal tract, and medial reticulospinal tract) WM tracts from the PAM50 atlas were combined [20]. The tracts were chosen to correspond to the assessed clinical scores in the ISNCSCI examination.

To capture lateralized recovery, WM tracts were divided into left and right sides. The extent of tract damage was quantified by calculating the percentage of overlap between the segmented lesion and the tract masks. Figure 1 illustrates a patient example of 3D tract damage analysis.

### 2.6 Lesion Parameters

TB were segmented on the midsagittal slice of a T2‐w scan, following previously reported methods [5, 6, 14]. Ventral and dorsal bridges were assessed separately and combined to calculate the total TB. Lesion volume was determined by manually delineating the lesion area on each sagittal slice of the spinal cord. The total lesion area was summed and multiplied by the sagittal scan's slice thickness to obtain the final lesion volume. Lesion positions on sagittal T2‐weighted MRI were categorized into four types based on the presence of intact TB: (1) centrally located, with both ventral and dorsal bridges intact; (2) ventrally located, with only dorsal bridges intact; (3) dorsally located, with only ventral bridges intact; and (4) complete, with no intact TB.

### 2.7 Statistical Analysis

All statistical analyses were conducted using R (version 4.3.1).

Sensory and motor tract damage across different lesion types (central, ventral, dorsal, and complete) was compared using independent samples two‐tailed *t*‐tests.

The predictive value of early tract damage for later clinical recovery was evaluated using a linear mixed model. Left and right motor tract damage at 1‐month post‐injury were used to predict left and right motor score changes at 6‐month, while left and right sensory tract damage at 1‐month was used to predict left and right pinprick score changes at 6‐month.

MRI parameters and clinical outcomes at 6‐month post‐injury were compared using a general linear model. Relationships between tract damage and corresponding clinical scores, as well as 2D TB and lesion volume, were assessed. MRI parameters and clinical scores at 6 months were selected for this analysis, as they stabilize by then.

Demographic and clinical variables were presented as means with 95% CI. All statistical tests were corrected for age, sex, and center as covariates of no interest, and only results with a significance threshold of *p* < 0.05 were reported.

## 3 Results

At baseline, SCI patients had a total mean motor score of 31.82 (95% CI: 25.63–38.01), which improved over time by 4.95/month (95% CI: 3.89–5.89, *p* < 0.001). Light touch, with a mean baseline score of 60.59 (95% CI: 67.03–83.35), improved by 2.28/month (95% CI: 1.43–3.12, *p* < 0.001). Pinprick, with a mean baseline score of 34.81 (95% CI: 25.56–44.07), improved by 2.06/month (95% CI: 1.21–2.91, *p* < 0.001).

At baseline, mean lesion volume was 381.82 mm^3^ (95% CI: 295.78–467.87), decreasing by −14.04 mm^3^/month (95% CI: −25.39 to −1.56, *p* = 0.023). Sensory tract damage, with a baseline mean of 69.57% (95% CI: 66.04–73.11), decreased by −0.69%/month (95% CI: −1.29 to 0.09, *p* = 0.05), while motor tract damage, at a baseline mean of 63.07% (95% CI: 57.86–68.28), showed no significant change, with a rate of 0.02%/month (95% CI: −0.81 to 1.02, *p* = 0.97) (Table 2). Larger sensory tract damage was observed at baseline compared to motor tract damage (*p* = 0.049).

**TABLE 2:** Linear mixed‐effects model to illustrate structural changes of the tract damage and clinical scores over 6 months.

Outcome variable	Standardized regression coefficient (95% CI)	Number of patients	*p*	*R* ^2^ marginal	*R* ^2^ conditional
Volume	−0.47 (−0.085 to −0.005)	35	0.02	0.15	0.56
Sensory tracts	−0.02 (−0.043 to 0.003)	35	0.05	0.28	0.57
Motor tracts	0.00 (0.027 to 0.034)	35	0.97	0.18	0.55
Motor score	0.16 (0.13 to 0.2)	35	< 0.001	0.22	0.86
Pinprick score	0.067 (0.04 to 0.1)	35	< 0.001	0.37	0.89
Light Touch score	0.08 (0.048 to 0.104)	35	< 0.001	0.26	0.87

*Note:* Age, sex, and center are put in the model as covariates of no interest.

**FIGURE 2:**
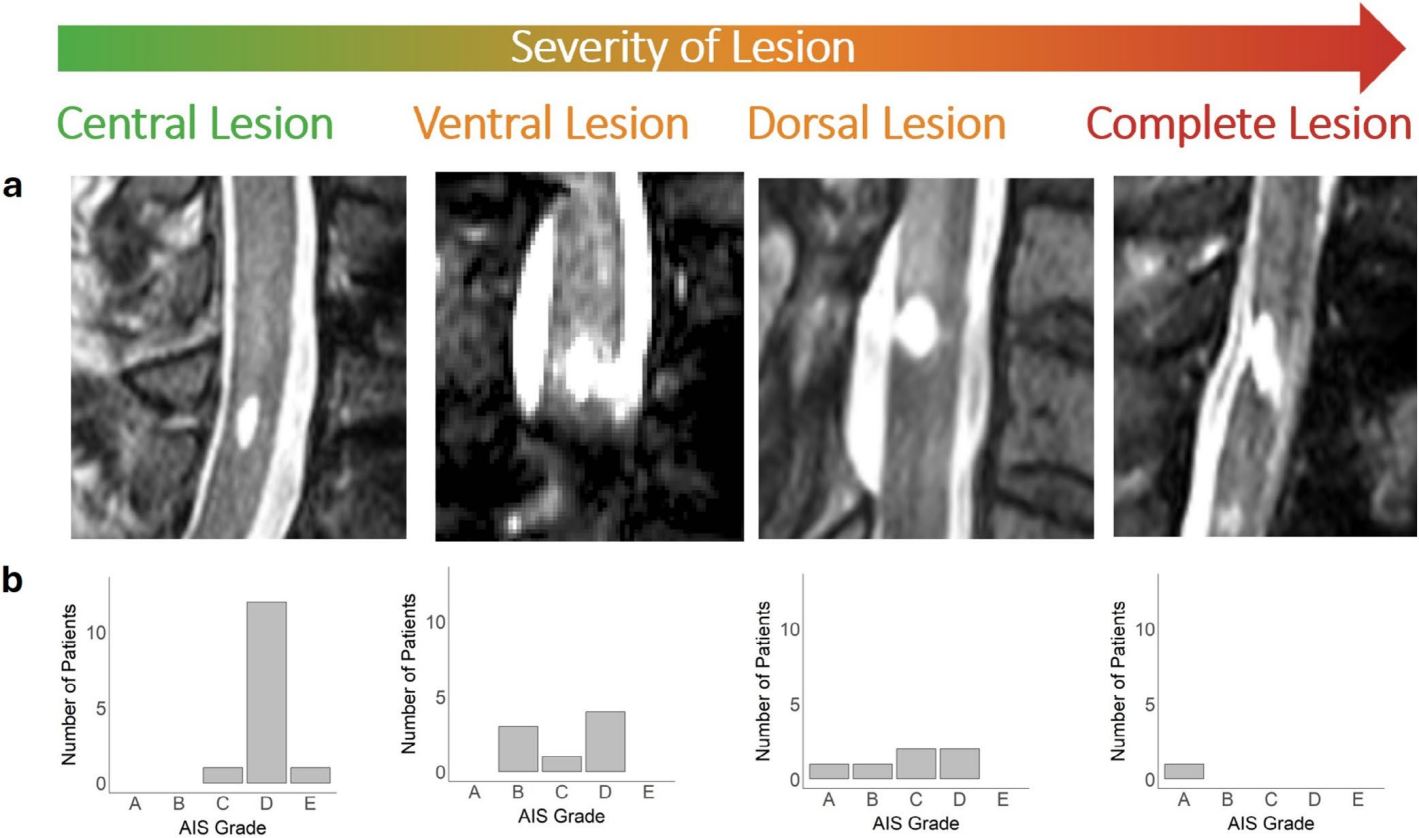
Lesion types identified on a midsagittal T2‐weighted MRI scan. (a) MRI scans illustrate the four lesion types: central, ventral, dorsal, and complete lesions (b) the corresponding bar plot below depicts the distribution of AIS grades for each lesion type, showing that centrally located lesions are generally associated with less impairment compared to complete lesions.

Figure 2 summarizes the distribution of AIS grades (AIS A to AIS D) across different lesion types. For centrally located lesions, sensory tract damage was 63.3% (95% CI: 59.5–67.2), significantly higher than motor tract damage, which was 55.2% (95% CI: 51.2–59.1; *p* = 0.004). In ventrally located lesions, sensory tract damage was slightly lower at 71.3% (95% CI: 67.0–75.5) compared to motor tract damage at 77.2% (95% CI: 72.7–81.7), with a trend toward significance (*p* = 0.051). For dorsally located lesions, sensory tract damage was 74.7% (95% CI: 70.2–79.1), which was significantly higher than motor tract damage at 65.5% (95% CI: 58.7–72.3; *p* = 0.023). In complete lesions, no significant difference was observed between sensory tract damage (74.2%, 95% CI: 67.4–81.0) and motor tract damage (71.6%, 95% CI: 63.2–80.1; *p* = 0.610).

**FIGURE 3:**
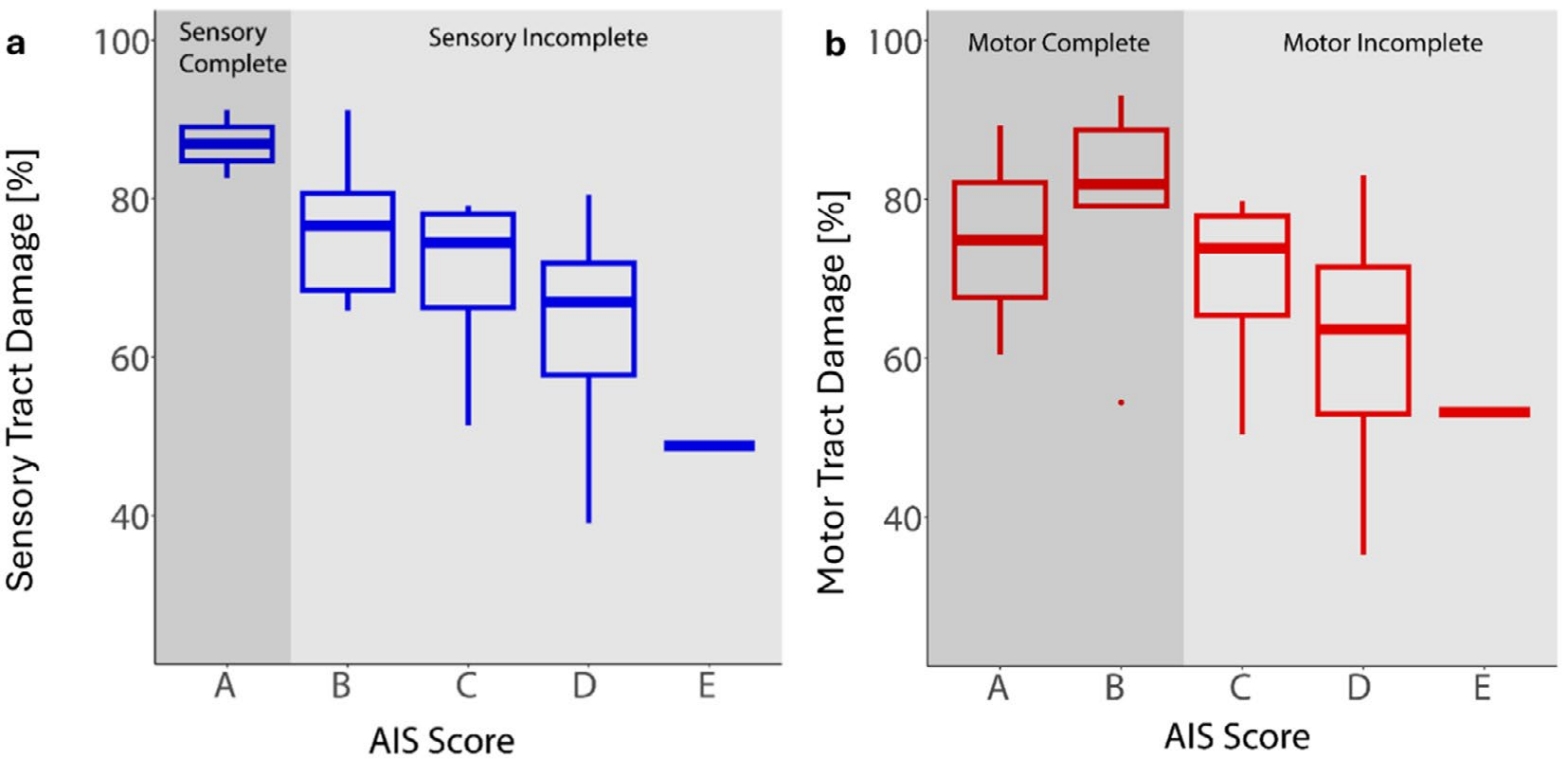
Box plots illustrating the relationship between AIS scores and tract damage at the 6‐month time point. (a) Sensory Tract Damage [%] by AIS Score. AIS A patients have the highest damage, followed by AIS B, C, D, and E in decreasing order. This shows the severity of sensory tract damage is highest in AIS A patients and decreases with lower AIS scores. (b) Motor Tract Damage [%] by AIS Score. AIS A and AIS B patients show the greatest damage, with AIS B slightly higher than AIS A. Damage decreases for AIS C, D, and E, reflecting the severity of the injury on motor pathways.

Figure 3 illustrates the 3D tract damage analysis, showing that sensory tract damage is most pronounced in the most impaired patients. Sensory and motor complete patients (AIS A) exhibited the highest sensory tract damage at 86.92% (95% CI: 32.64–141.20), followed by a gradual decrease with improving AIS grades: AIS B at 76.33% (95% CI: 66.22–86.43), AIS C at 69.85% (95% CI: 49.48–90.21), AIS D at 64.51% (95% CI: 59.31–69.72), and AIS E at 48.80%. Motor tract damage was greatest in motor complete patients, with AIS A at 74.86% (95% CI: −108.08–257.81) and AIS B at 80.10% (95% CI: 65.64–94.56). For motor incomplete patients, motor tract damage decreased in line with AIS grades: AIS C at 69.47% (95% CI: 48.30–90.63), AIS D at 61.58% (95% CI: 55.25–67.91), and AIS E at 53.21%.

Left and right motor tract damage at 1‐month post‐injury predicted left and right motor score changes at 6‐month (*p* = 0.015, *R* ^2^ = 0.75) and left and right sensory tract damage at 1‐month predicted bilateral pinprick score changes at 6‐month (*p* = 0.024, *R* ^2^ = 0.79) (Table 3, Figure S2).

**TABLE 3:** Linear mixed‐effects model to predict left and right clinical score changes from 1‐ to 6‐month post‐injury with left and right tract damage at 1‐month post‐injury.

Outcome variable	Clinical score changes	Number of patients	Standardized regression coefficient (95% CI)	*p*	*R* ^2^ marginal	*R* ^2^ conditional
Motor tracts	Motor score	27	−0.28 (−0.52 to −0.08)	0.015	0.16	0.75
Sensory tracts	Pinprick score	27	−0.19 (−0.44 to −0.06)	0.024	0.45	0.79

*Note:* Age, sex, and center were included as covariates of no interest.

3D tract‐specific damage at 6‐month post‐injury showed associations to motor scores (*p* = 0.0026, *R* ^2^ = 0.29), pinprick (*p* = 0.035, *R* ^2^ = 0.61) and light touch score (*p* = 0.007, *R* ^2^ = 0.57) at 6‐month post‐injury. TB at 6‐month post‐injury showed associations to motor scores (*p* = 0.003, *R* ^2^ = 0.28), pinprick (*p* = 0.020, *R* ^2^ = 0.63) and light touch score (*p* = 0.010, *R* ^2^ = 0.56) at 6‐month post‐injury. Lesion volume at 6‐month post‐injury showed associations to motor scores (*p* = 0.004, *R* ^2^ = 0.27) and pinprick (*p* = 0.013, *R* ^2^ = 0.65) at 6‐month post‐injury (Table 4, Figure S3).

**TABLE 4:** Linear model to illustrate correlation of the 6‐month time point MRI parameters and 6‐month time point clinical scores.

Outcome variable	Clinical score	Number of patients	Standardized regression coefficient (95% CI)	*p*	*R* ^2^	*R* ^2^ adjusted
Motor tracts	Motor score	33	−0.55 (−0.9 to −0.21)	0.0026	0.29	0.21
Sensory tracts	Pinprick score	33	−0.42 (−0.81 to −0.33)	0.035	0.61	0.40
Sensory tracts	Light touch score	33	−0.58 (−0.99 to −0.18)	0.007	0.57	0.34
Tissue bridges	Motor score	33	0.55 (0.20 to 0.90)	0.0031	0.28	0.20
Tissue bridges	Pinprick score	33	0.40 (0.07 to 0.73)	0.020	0.63	0.43
Tissue bridges	Light touch score	33	0.48 (0.13 to 0.85)	0.010	0.56	0.32
Lesion volume	Motor score	33	−0.57 (−0.94 to −0.20)	0.0035	0.27	0.19
Lesion volume	Pinprick score	33	−0.65 (−0.80 to −0.11)	0.013	0.65	0.45
Lesion volume	Light Touch	33	−0.40 (−0.82 to 0.018)	0.06	0.48	0.20

*Note:* Age, sex, and center are put in the model as covariates of no interest.

## 4 Discussion

This methodolgical study shows that the 3D evaluation of motor and sensory‐specific tract damage at 1 month post‐injury is highly predictive of related clinical recovery. This detailed 3D assessment of spinal lesions offers insights into the impact on recovery that goes beyond traditional 2D analyses, allowing for a comprehensive evaluation of the entire lesion.

As anticipated, acute SCI patients show some extent of clinical recovery during the study in both motor and sensory scores, consistent with established recovery trajectories [21, 22]. Concurrently, the lesion size shrank significantly post‐SCI, which aligns with previous findings [4]. Lesion location analysis in the sagittal plane highlights that centrally located lesions, which preserve both ventral and dorsal TB, are associated with less severe clinical impairment, as reflected by AIS grades. This was to be expected, as earlier studies demonstrated that the width of TB plays a critical role in clinical recovery after cervical SCI [6, 7, 14]. Extending this analysis to 3D tract damage provides additional insights, with the extent of sensory and motor tract damage at 6 months post‐injury closely tied to AIS grades. Patients with AIS A exhibit the most severe tract damage, particularly in the sensory tracts, corresponding to complete sensory deficits. Likewise, motor complete patients demonstrate greater motor tract damage, with AIS B showing slightly more extensive damage than AIS A.

Examining the four lesion types and their relation to tract damage reveals that ventral lesions are associated with greater motor tract damage, while dorsal lesions result in more extensive sensory tract damage. Complete lesions, on the other hand, showed no difference in damage extent for motor and sensory tracts. Notably, central lesions exhibit significantly greater sensory tract damage compared to motor tract damage. This suggests that centrally located lesions may have a disproportionate effect on sensory pathways. This pattern of disproportionate sensory tract involvement becomes even more evident upon closer 3D examination of tract‐specific damage. Sensory tracts, which were more severely damaged at baseline, exhibited greater changes in lesion extent over time compared to motor tracts. This suggests that the dorsal region of the spinal cord undergoes more dynamic alterations within the first 6 months after injury [23]. These MRI‐visible changes can be attributed to Wallerian degeneration in the dorsal region, for example, sensory tracts, which occurs earlier after injury and affects a larger area compared to motor tracts [24, 25, 26]. The dynamic nature of lesion evolution, particularly the varying responses of different tracts, highlights the importance of 3D spatiotemporal analysis in enhancing our understanding of lesion progression.

Crucially, at 1‐month post‐injury, left and right sensory and motor tract damage was found to predict changes in left and right pinprick and motor scores, respectively. Conventional 2D lesion assessments, such as TB, cannot provide this kind of insight because they span the anterior–posterior direction and lack information about left–right asymmetry [5, 6, 9, 14, 27]. No significant association was found between sensory tract damage and light touch scores over time, nor was it predictive of recovery of light touch. The light touch test primarily assesses the integrity of the dorsal columns, while the pinprick test, which evaluates the ability to differentiate between sharp and dull sensations, activates the spinothalamic tract [17, 28]. An earlier study has shown that dorsal column damage predicts light touch recovery [10]. In this study, however, the resolution of the sagittal MRI does not yet allow for the assessment of damage to individual tracts, which is an inherent limitation. Moreover, spinal cord imaging remains inherently challenging compared to brain imaging due to physiological confounders like respiratory motion, aortic blood flow, and magnetic susceptibility differences between surrounding tissues, which can introduce artifacts and reduce image quality [29, 30]. These factors limit the precise visualization of individual tracts and their damage. Future work will focus on utilizing higher‐resolution sagittal scans to enable more detailed segmentation and analysis of tract‐specific damage.

At 6 months post‐injury, sensory tract damage was significantly correlated with light touch and pinprick scores on the ISNCSCI exam at the same time point, and motor tract damage was associated with motor score impairments. Both 2D midsagittal TB and lesion volume also revealed associations with clinical scores at 6 months, indicating that conventional lesion assessments effectively explain variations in clinical outcomes during this period, confirming results from earlier studies [4, 5, 6, 9]. However, TB primarily assess the dorsal and ventral columns on the midsagittal slice, potentially missing the integrity of spinothalamic tracts related to pinprick scores, highlighting limitations in this approach. A more comprehensive injury assessment is achieved through lesion volume, which includes data from all slices, accounting for lateral injury differences. Previous studies have shown lesion volume's improved prognostic capability compared to traditional 2D parameters like lesion length and BASIC score [27]. Lesion volume alone may oversimplify spinal injury, as two patients with similar volumes can exhibit different injury patterns, for example, varying tract damage, leading to differing recovery potentials. Including lesion localization within the spinal cord, as demonstrated in the 3D tract damage assessment, can provide a more precise insight into clinical recovery.

This study has several limitations. The cohort with 35 patients is relatively small, and only cervical SCI patients were part of the study. This leads to a relatively homogeneous sample but may not reflect the general SCI population, and the findings might not be transferable to paraplegic patients [3]. The cohort was predominantly male, consistent with the general sex distribution observed in the SCI population, and is not expected to significantly impact the findings [31]. The resolution varies between subjects with sagittal slice thickness, ranging from 2.2 to 4.4 mm (Table S1). However, no correlation (*p* = 0.88) between lesion extent and slice thickness was found. Transforming to the axial plane introduces an extra processing step, which may introduce errors. However, even with this added step, more variance was explained by the sensory and motor tract damages than conventional 2D lesion assessments, as indicated by higher *R* ^2^ values, along with improved sensitivity to lateralized clinical improvements. Additionally, our approach allowed for a direct comparison with TB and lesion volume, as the segmentation was performed on the same sagittal scans.

## 5 Conclusion

This study shows the dynamics of 3D tract‐specific damage in cervical SCI, explored through longitudinal MRI assessments during the first 6 months post‐injury. By mapping sagittal lesion segmentations onto the axial plane, the analysis of sensory and motor white matter tract damage was conducted using conventional clinical MRI scans acquired at the lesion site. This approach allows for a detailed comparison of lesion evolution and its impact on lateralized functional outcomes, such as left and right motor and sensory recovery. While correlations at 6 months post‐injury between 3D tract‐specific damage and clinical scores are similar to those seen with conventional lesion assessments, such as 2D midsagittal TB and lesion volume, tract‐specific analysis provides the added benefit of a 3D evaluation, assessing left and right‐sided damage separately. This makes it a more relevant predictive neuroimaging biomarker, offering valuable insights for personalized treatment strategies and patient stratification in both acute and chronic clinical trials, using routine clinical MRI scans.

## Author Contributions

L.F. and T.M.E. contributed to the conception, methodology, and analysis of the manuscript. M.S., A.C., and P.F. were responsible for data acquisition. L.F. drafted the original manuscript. L.F., T.M.E., S.S.S., J.B., M.S., A.C., and P.F. contributed to the review and editing of the manuscript.

## Conflicts of Interest

The authors declare no conflicts of interest.

## Supporting information


**Figure S1:** Study profile.
**Figure S2:** Depiction of predictive correlations between 3D tract damage measured at the 1‐month time point and changes in clinical scores between the baseline and 6‐month time point. The left panel shows a significant negative correlation between motor tract damage and changes in motor scores, suggesting that greater motor tract damage at 1 month is associated with reduced motor recovery at 6 months. The right panel displays a negative correlation between sensory tract damage and changes in pinprick scores, indicating that more severe sensory tract damage at 1 month is associated with less improvement in pinprick scores over 6 months.
**Figure S3:** Illustration of the correlations between 3D tract damage and 2D tissue bridges and lesion volume and normalized clinical scores (motor, pinprick, and light touch) assessed at the 6‐month time point. (a) Correlations for motor scores, showing negative associations with lesion volume and motor tract damage, and a positive association with midsagittal tissue bridges. (b) Correlations for pinprick scores, revealing similar trends: a negative relationship with lesion volume and sensory tract damage, and a positive correlation with midsagittal tissue bridges. (c) Light touch scores, which also demonstrate negative correlations with lesion volume and sensory tract damage, and a positive correlation with midsagittal tissue bridges.
**Table S1:** MRI parameters for all patients for all three time points. The parameters include the slice thickness, the in‐plane resolution, the repetition time (TR), the echo time (TE), the flip angle (FA), and the strength of the MR magnet (field strength). The centers involved in the study are Barcelona (BCA), Bochum (BCM), Basel (BSL), Bayreuth (BYH), Heidelberg (HDG), Halle (HLE), Murnau (MNU), Nottwil (NTL) and Zurich (ZRH).
**Table S2:** Linear model to illustrate correlation of the 6‐month time point MRI parameters and 6‐month time point total clinical scores. Age, sex, and center are put in the model as covariates of no interest.

Supinfo2.

## Data Availability

Anonymized data not published within this article will be made available by request from any qualified investigator.
