# Supplementary material for: 3D MRI Tract‐Specific Spinal Cord Lesion Pattern Improves Prediction of Distinct Neurological Recovery
Source: Ann Clin Transl Neurol. 2025 Jun 27;12(9):1762–70. doi: 10.1002/acn3.70087 (PMC12455861; doi:10.1002/acn3.70087)
Supplement: Supplementary file 1 — Figure S1: Study profile. Figure S2: Depiction of predictive correlations between 3D tract damage measured at the 1‐month time point and changes in clinical scores between the baseline and 6‐month time point. The left panel shows a significant negative correlation between motor tract damage and changes in motor scores, suggesting that greater motor tract damage at 1 month is associated with reduced motor recovery at 6 months. The right panel displays a negative correlation between sensory tract damage and changes in pinprick scores, indicating that more severe sensory tract damage at 1 month is associated with less improvement in pinprick scores over 6 months. Figure S3: Illustration of the correlations between 3D tract damage and 2D tissue bridges and lesion volume and normalized clinical scores (motor, pinprick, and light touch) assessed at the 6‐month time point. (a) Correlations for motor scores, showing negative associations with lesion volume and motor tract damage, and a positive association with midsagittal tissue bridges. (b) Correlations for pinprick scores, revealing similar trends: a negative relationship with lesion volume and sensory tract damage, and a positive correlation with midsagittal tissue bridges. (c) Light touch scores, which also demonstrate negative correlations with lesion volume and sensory tract damage, and a positive correlation with midsagittal tissue bridges. Table S1: MRI parameters for all patients for all three time points. The parameters include the slice thickness, the in‐plane resolution, the repetition time (TR), the echo time (TE), the flip angle (FA), and the strength of the MR magnet (field strength). The centers involved in the study are Barcelona (BCA), Bochum (BCM), Basel (BSL), Bayreuth (BYH), Heidelberg (HDG), Halle (HLE), Murnau (MNU), Nottwil (NTL) and Zurich (ZRH). Table S2: Linear model to illustrate correlation of the 6‐month time point MRI parameters and 6‐month time point total clinical scores. Age, sex, and cent [file ACN3-12-1762-s001.docx]

**Supplementary Appendix**

This appendix has been provided by the authors to give readers additional information about their work. Supplement to: Farner et al., 3D MRI tract-specific spinal cord lesion pattern improve prediction of distinct neurological recovery: Insights from a multicenter study

**Supplementary Table 1** MRI parameters for all patients for all three time points. The parameters include the slice thickness, the in-plane resolution, the repetition time (TR), the echo time (TE), the flip angle (FA), and the strength of the MR magnet (field strength). The centers involved in the study are Barcelona (BCA), Bochum (BCM), Basel (BSL), Bayreuth (BYH), Heidelberg (HDG), Halle (HLE), Murnau (MNU), Nottwil (NTL) and Zurich (ZRH).

| **Slice Thickness [mm]** | **In plane resolution [mm]** | **TR [ms]** | **TE [ms]** | **FA [°]** | **MR Field strength [T]** | **Center** | **Scanner** | **Number of Patients** |
| --- | --- | --- | --- | --- | --- | --- | --- | --- |
| 2.75 | 0.34 x 0.34 | 4240 | 84 | 124-160 | 3 | BCA | Siemens Verio | 1 |
| 2.75 | 0.34 x 0.34 | 3500 | 84 | 160 | 1.5/3 | BCM | Siemens Skyra | 2 |
| 2.75 | 0.25 x 0.35 | 3500 | 80 | 160 | 3 | BSL | Siemens Prisma | 1 |
| 3.3-4.4 | 0.54-0.81 x 0.54-0.81 | 3150-4370 | 75-108 | 150-160 | 3 | BYH | Siemens Skyra | 6 |
| 2.75 | 0.69-0.78 x 0.69-0.78 | 3500 | 75 | 160 | 3 | HDG | Siemens Verio | 7 |
| 2-2.75 | 0.37-0.52 x 0.69-0.78 | 1200-3313 | 80-150 | 90 | 3 | HLE | Philips Ingenia | 3 |
| 2.75 | 0.44 x 0.44 | 3000-3216 | 80 | 90 | 3 | MNU | Philips Achieva | 6 |
| 2.75 | 0.44 x 0.44 | 3000 | 80 | 90 | 3 | NTL | Philips Achieva | 5 |
| 2.75 | 0.34-0.57 x 0.34-0.57 | 3500-3760 | 84-91 | 136-160 | 3 | ZRH | Siemens Skyra | 4 |

**Supplementary Figure 1** Study Profile


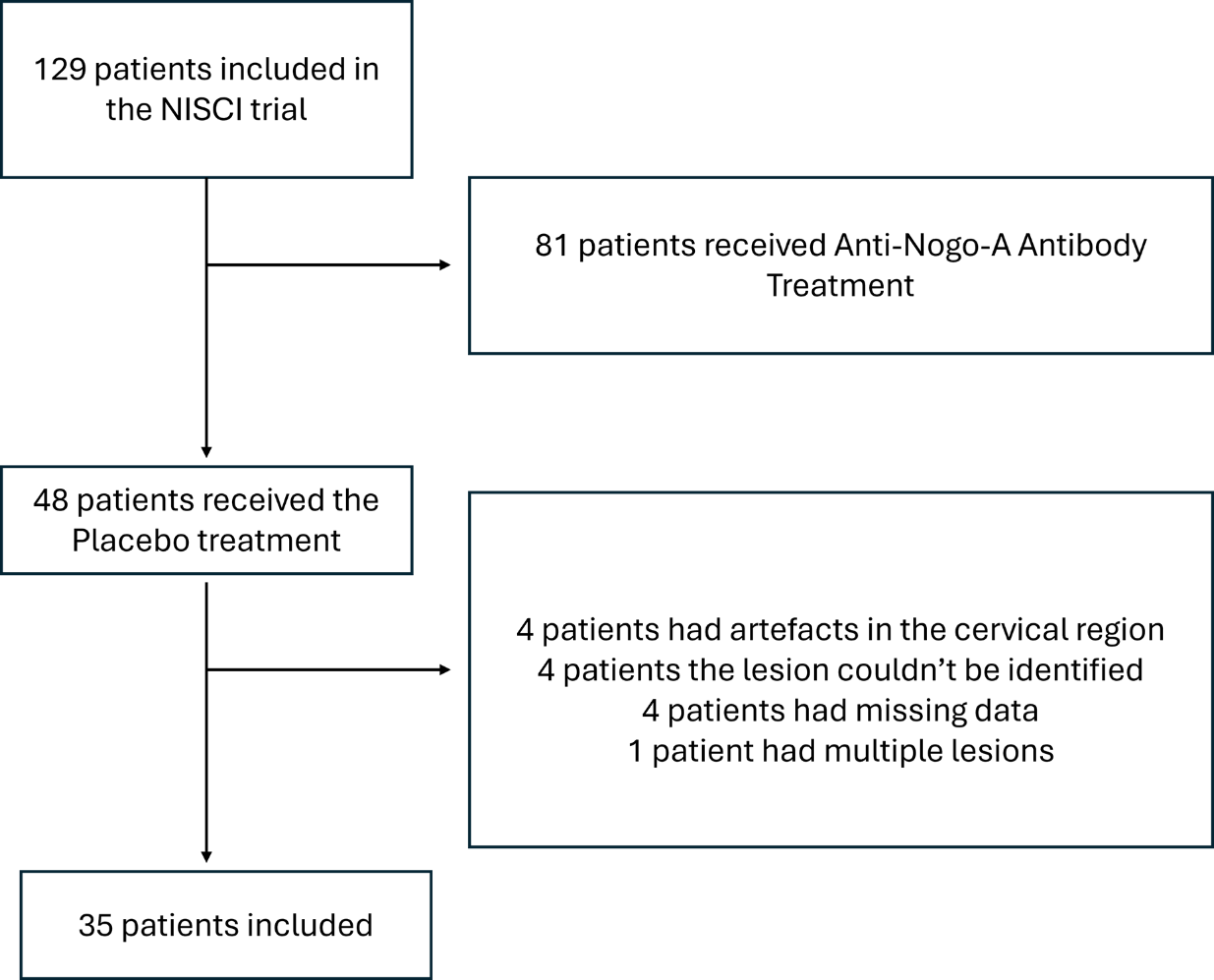


**Supplementary Figure 2** Depiction of predictive correlations between 3D tract damage measured at the 1-month time point and changes in clinical scores between the baseline and 6-month time point. The left panel shows a significant negative correlation between motor tract damage and changes in motor scores, suggesting that greater motor tract damage at 1 month is associated with reduced motor recovery at 6 months. The right panel displays a negative correlation between sensory tract damage and changes in pinprick scores, indicating that more severe sensory tract damage at 1 month is associated with less improvement in pinprick scores over 6 months.


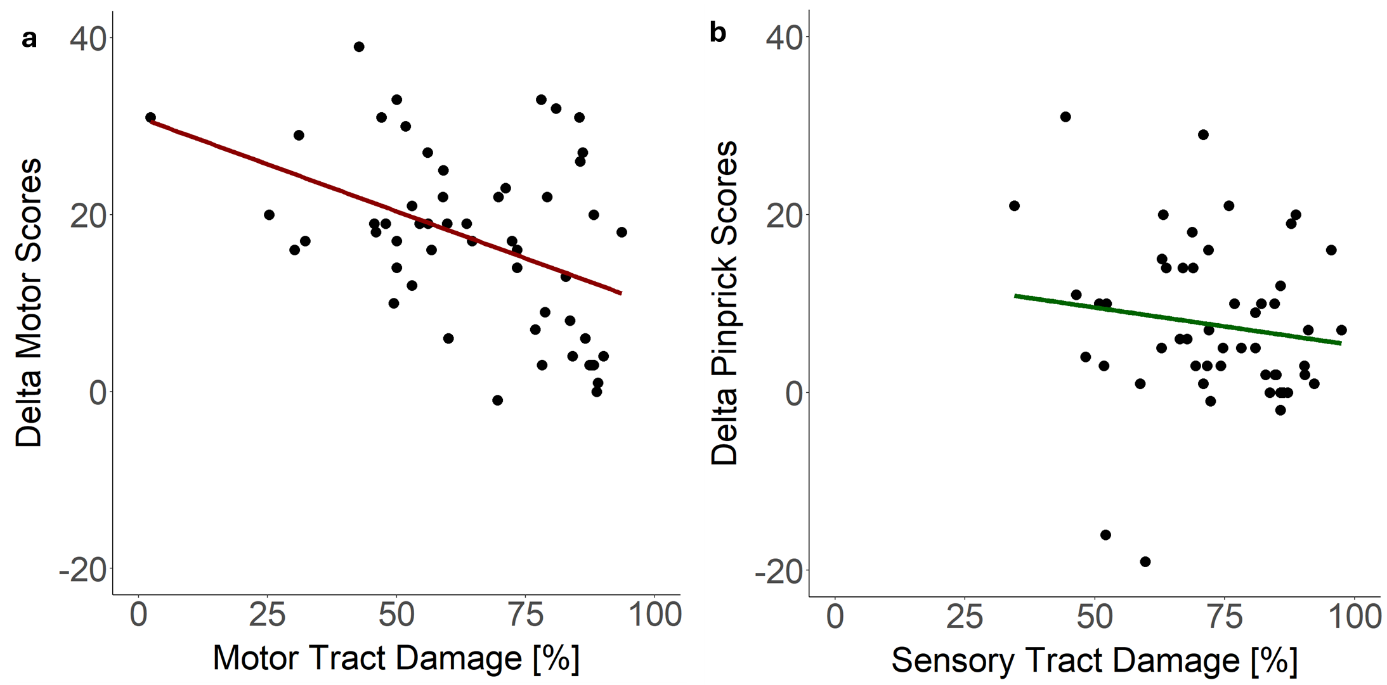


**Supplementary Figure 3** Illustration of the correlations between 3D tract damage and 2D tissue bridges and lesion volume and normalized clinical scores (motor, pinprick, and light touch) assessed at the 6-month time point. a) correlations for motor scores, showing negative associations with lesion volume and motor tract damage, and a positive association with midsagittal tissue bridges. b) Correlations for pinprick scores, revealing similar trends: a negative relationship with lesion volume and sensory tract damage, and a positive correlation with midsagittal tissue bridges. c) light touch scores, which also demonstrate negative correlations with lesion volume and sensory tract damage, and a positive correlation with midsagittal tissue bridges.


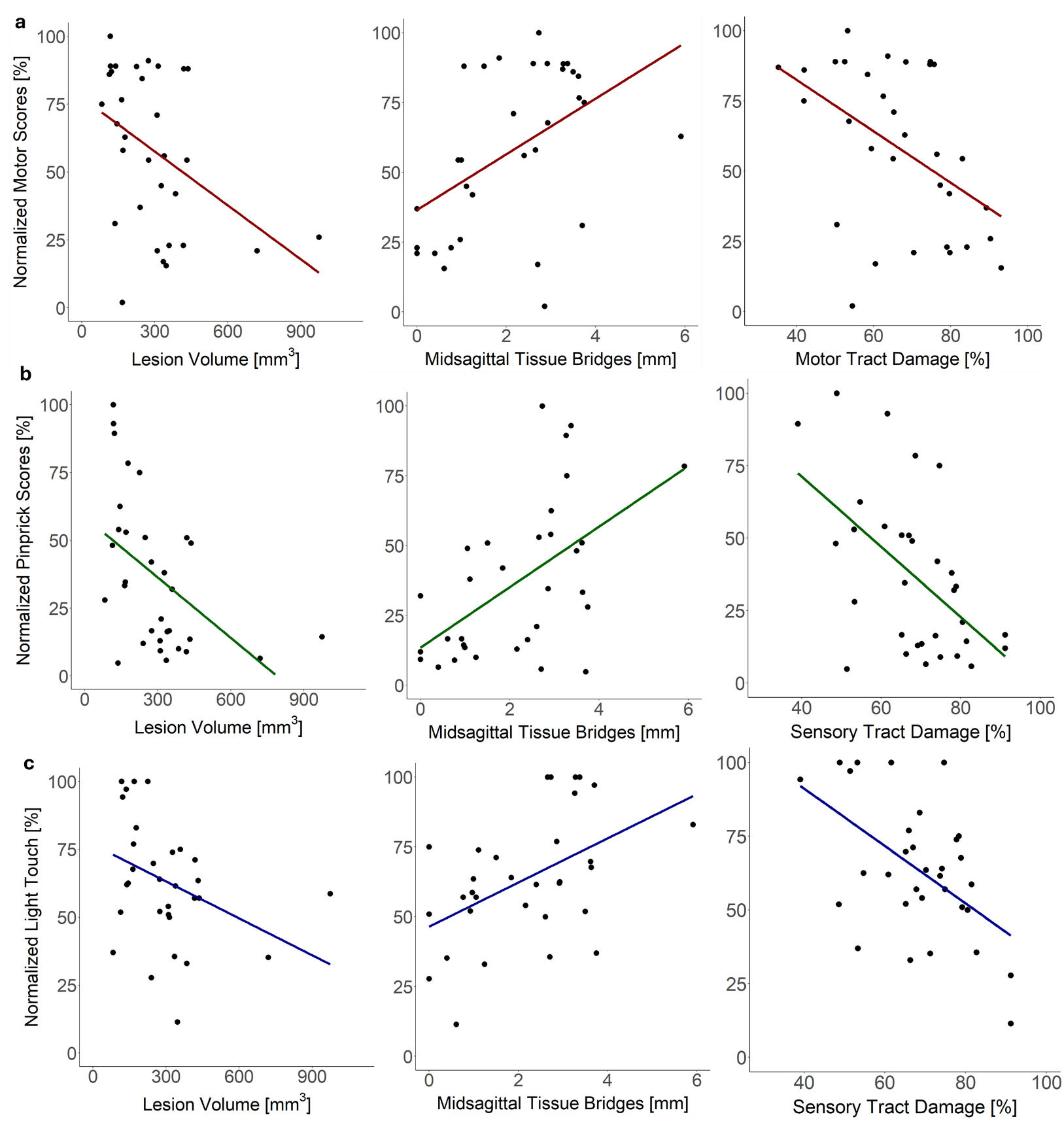


**Supplementary Table 2** Linear model to illustrate correlation of the 6 -month time-point MRI parameters and 6-month time-point total clinical scores. Age, sex, and center are put in the model as covariates of no interest.

| **Outcome Variable** | **Clinical Score** | **Number of Patients** | **Standardized Regression Coefficient (95% CI)** | **p-value** | **R^2^** | **R^2^ adjusted** |
| --- | --- | --- | --- | --- | --- | --- |
| Motor tracts | Motor score | 33 | -0.51 (-0.87 to -0.17) | 0.0046 | 0.26 | 0.18 |
| Sensory tracts | Pinprick score | 33 | -0.43 (-0.83 to -0.03) | 0.036 | 0.59 | 0.37 |
| Sensory tracts | Light touch score | 33 | -0.60 (-1.01 to -0.19) | 0.0065 | 0.57 | 0.33 |
| Tissue bridges | Motor score | 33 | 0.57 (0.23 to 0.92) | 0.002 | 0.30 | 0.22 |
| Tissue bridges | Pinprick score | 33 | 0.42 (0.09 to 0.76) | 0.015 | 0.62 | 0.41 |
| Tissue bridges | Light touch score | 33 | 0.51 (0.16 to 0.87) | 0.007 | 0.56 | 0.32 |
| Lesion volume | Motor score | 33 | -0.58 (-0.94 to -0.21) | 0.003 | 0.28 | 0.20 |
| Lesion volume | Pinprick score | 33 | -0.47 (-0.83 to -0.12) | 0.011 | 0.63 | 0.43 |
| Lesion volume | Light Touch | 33 | -0.42 (-0.84 to 0.01) | 0.053 | 0.48 | 0.19 |
