# Supplementary material for: 3D MRI Tract‐Specific Spinal Cord Lesion Pattern Improves Prediction of Distinct Neurological Recovery
Source: Ann Clin Transl Neurol. 2025 Jun 27;12(9):1762–70. doi: 10.1002/acn3.70087 (PMC12455861; doi:10.1002/acn3.70087)
Supplement: Supplementary file 2 — Supinfo2. [file ACN3-12-1762-s002.pdf]

Study Personnel: The Nogo Inhibition in Spinal Cord Injury Study Group and site study personnel

Germany
Bayreuth: Rainer Abel M.D., Katja-Kerstin Bössl M.D., Anika Steger,

Berlin: Sandra Arndt, Malte Gössling M.D., Martin Kreuzträger M.D., Thomas Liebscher M.D., Franziska Radach, Kerstin Rehahn M.D.

Bochum: Mirko Aach M.D., Katalin Barkovits, Ph.D., Dennis Grasmücke M.D., Manuela Klettke, Katrin, Marcus Ph.D.

Halle: Gudrun Allmendinger, Carolin Gräbsch, Frank Röhrich M.D., Klaus Röhl M.D., F. W. Weidt M.D.

Heidelberg: Ina Burghaus Ph.D., Stefan Fichtner, Steffen Franz M.D., Cornelia Hensel M.D., Laura Heutehaus M.Sc., Johannes Hüsing Ph.D., Andreas Hug M.D., Naemi Kühn Ph.D., Annette Langpape, Antonia- Sophie Luz, Melanie Motsch, Radhika Puttagunta Ph.D., Christoph Rehnitz M.D., Rüdiger Rupp Ph.D., Christian Schuld M.Sc., Dominik Teichert Ph.D., Björn Wagner M.D., Norbert Weidner M.D., Tanja Weis Ph.D.

Hessisch-Lichtenau:Nadine Rohleder, Marion Saur M.D., Josina Waldmann M.D.

Leipzig: Tobias Leutritz Ph.D., Nikolaus Weiskopf Ph.D.

Murnau: Lukas Grassner M.D., Orpheus Mach, Doris Maier M.D., Ludwig Sanktjohanser M.D., Matthias Vogel M.D.,Tübingen Andreas Badke M.D., Elke Kirsch, Marie Reumann M.D.

Switzerland
Basel: Vera Bouverat M.D., Isabelle Debecker, Kerstin Hug M.D., Margret Hund-Georgiadis M.D., Holger Lochmann M.D., Elena Pauli

Bern: Petra Zalud, Ph.D.

Nottwil: Michael Baumberger M.D., Desiree Beck, Agata Bulloni, Angela Frotzler Ph.D., Evelyn Rickenbacher, Anke Scheel M.D., Jürgen Schneider M.D.

Zürich: Marc Bolliger Ph.D., Armin Curt M.D., Lynn Farner M.Sc., Patrick Freund M.D., Torsten Hothorn Ph.D., Michèle Hubli Ph.D., Tim Killeen M.D., Iris Krüsi, Michael A. Maurer Ph.D., Andrea Prusse, Paulina S. Scheuren Ph.D., Martin Schubert M.D., Martin E. Schwab Ph.D., Maryam Seif, Christina, Sina Ph.D., Bettina Steiner M.Sc.

Spain
Barcelona: Jesus Benito-Penalva M.D., Hatice Kumru M.D., Josep Medina Ph.D., Eloy Opisso Ph.D., Joan Vidal M.D.,

Czechia
Prague:Renata Hakova M.D., Veronika Hysperska M.D., Jiri Kriz M.D.
